# Collecting grief: Indigenous peoples, deaths by police and a global pandemic

**DOI:** 10.1177/1473325020973301

**Published:** 2021-03

**Authors:** Mary Kate Dennis

**Affiliations:** Faculty of Social Work, University of Manitoba, Winnipeg, Canada

**Keywords:** Indigenous, loss, death, violence

For centuries, Indigenous people in North American have endured a vast array of interruptions and disruptions affecting all aspects of our culture and societies. As a result, Indigenous people have had to respond by continuously adapting traditional ways and have grieved these losses individually and communally. The ongoing conditions directly associated with settler colonialism are revealed in systematic and systemic racism and violence in the present. The global COVID-19 pandemic revealed more publically these structural inequities that are experienced by Indigenous people living in Winnipeg, Manitoba, Canada. In April 2020, three Indigenous people were killed by police, causing Indigenous people to adapt the ways they participate in end-of-life ceremonies. Through our communal connections, when something happens to one Indigenous person, it happens to all of us. As the community addresses these losses, they do so under the constraints of the pandemic and the systemic racism propagated through the colonial lens of the police and the news media which continues to misunderstand and critique Indigenous people’s ways. This essay will explore the ways in which grieving is a communal experience for Indigenous people and how the global pandemic COVID-19 further complicates grieving for Indigenous peoples.

## Background

In April 2020, over the span of 10 days, three Indigenous people were killed by Winnipeg police officers in three separate incidents. They are Eishia Hudson (age 16), Jason Collins (age 36) and Stewart Kevin Andrews (age 22) (Berman, 2020). Unfortunately, untimely deaths caused by police violence are all too familiar in Indigenous communities. In Manitoba, Indigenous people form 15% of the population but represent 62% of the deaths by police (Palmater, 2020). Because of this, when the news notifications stated that a 16-year-old girl was killed by police, I knew she was Indigenous.

As Indigenous people are faced with the injustices surrounding these deaths, surviving loved ones are thrown into disarray as their ability to participate in end-of-life ceremonies are impeded. One way that Indigenous people have adapted is to gather to publically address these tragedies while simultaneously fostering support from one another as they begin to grieve communally. Adhering to social restrictions, a “Justice for Eishia Hudson Car Vigil” occurred on April 12, 2020, with people gathering to leave mementos, grieve communally and demand justice, which all occurred at the site where she was shot by police (Hatherly, 2020). The local media labeled the participants as protesters (Winnipeg Police (@wpgpolice), 2020a) misunderstanding and detracting from their central message. Additionally, police blocked the intersection, citing safety precautions, essentially making the statement that traffic or other peoples’ priorities needed to take precedence over communal grieving (Winnipeg Police (@wpgpolice), 2020b), whereas the news media (Hatherly, 2020) and a community member expressed via Twitter, that the police intervention “actively blockaded people similarly from mourning Eisha Hudson” (sic) (Green, 2020). Again, Indigenous people are prevented from participation, causing further adaptation and attempting to grieve in the face of adversity.

## Indigenous research

At the time of these incidents, I was conducting research focusing on Indigenous loss and grief. Building upon the knowledge shared by Lakota elders, I am continuing my research by developing relationships with Indigenous communities and Knowledge Holders/Elders in Manitoba. The Indigenous peoples in Manitoba include: Cree, Ojibwe, Dene, Oji-Cree, Dakota and Metis. In addition, I am a faculty member in the Master of Social Work based in Indigenous Knowledges (MSW-IK) Program at the University of Manitoba, where we foster anticolonialism and social work practices through Indigenous lenses. All of our courses are co-taught with a Cree and an Ojibwe Knowledge Holder. Through these relationships, I have the opportunity to explore the similarities and differences across the cultural groups in Manitoba and in relation to my own Indigeneity and the knowledge gained from my research.

In my research collaboration with Lakota elders, they shared with me the philosophy of *mitakuye oyasin,* which is simply translated as “we are all related” or “all my relations.” Albert White Hat (2012) states that this term is often used in Lakota communities and reveals Lakota philosophy, ways of life and explains their relationship to creation. Personally and professionally, I have come to know Indigenous knowledges, including the connections to creation and to each other, or *mitakuye oyasin,* are fostered in ceremony and rituals (White Hat, 2012). Ceremonies are designed to give us “strength, endurance, courage, or encouragement” (White Hat, 2012: 75). In the teachings shared by local Knowledge Holders, they offered insight into the similar philosophies of “we are all related” and the connections related to the creation stories for Cree and Ojibwe peoples. This philosophy helps us understand more fully the relationships that Indigenous people have with each other reinforced through ceremony.

## Indigenous spirituality and ceremony

Indigenous people are connected to each other and this is reinforced through our philosophies, values, spiritual beliefs and practices. It is difficult to write about Indigenous spirituality because it is something that is felt, experienced, and observed. Our culture and spirituality have been written about in a way that resulted in “mistranslation, misinterpretation, mystery and romance written about us” (White Hat, 2012). As a result, when discussing Indigenous spirituality and ceremony in a scholarly context, the Indigenous words used to fully express this lived experience does not translate well into English or Western intellectual frameworks. White Hat (2012) asserts that while we live in a modern context, “the spirituality, the life, is still there”, meaning that being Indigenous is both metaphysical and physical. The connection between and among Indigenous people is captured in that short sentiment – it’s still there, though capturing the full dynamics of a living universe in writing remains difficult.

White Hat (2012) offers insights into Lakota traditional beliefs and practices: there are unique and intricate ways that individuals, families and communities practice and use these ways to connect and support as they navigate loss and grief. White Hat offers examples to further understand a part of the intentions of practicing ceremony by asking the spirits for health and healing of a loved one. Spiritual support, the communal power of praying for and with one another is found by gathering, eating and telling stories at the ceremonies. The orientation towards others, our families, communities, the collective are central to our worldview and we ask the spirits, our relatives for help and support for what we as well as others may need help and support with when we pray.

There are practices related to death and burials for loss and grief. When someone dies, there are ceremonies to help the person’s spirit to “go on a good journey – not to look back, to have a good journey” (White Hat, 2012: 68). White Hat (2012) offers further insights into mourning related to Lakota people, both historical and contemporary. The periods of mourning focus on living and moving forward in a positive way. The community have a role in this process, by helping in preparation, organization, and throughout the ceremony. While some may envision a formal ceremony setting, we are often in ceremony the moment a relative dies until we can stop mourning. For the person who died, protocol instructs that their spirit has to move on to the next place in the journey, and there are a number of ways we are guided during this time; one way in particular is to not cry (White Hat, 2012). When we cry we hold them back, because we are expressing distress and suffering, so naturally a relative is empathetic and thus they suffer, and if the outcome is a desire for your loved one to be happy, then we have to let them go (White Hat, 2012). It should be noted that this is a traditional teaching and as Indigenous people adapt to modern contexts, we may not be able to uphold this teaching as we live very differently now than our ancestors did. As we face injustices and collect the layers of complications for the grief, we must be able to process these feelings as needed which likely involves crying. Memorial feasts mark the end of mourning, where families offer prayer and physical offerings to all of creation here and in the spirit world, with the intention of letting the spirit go (White Hat, 2012). Additionally, many tribes share a related philosophy with similar values and purposes that underlie their ceremony and rituals.

## Communal grief

The first identified case of COVID-19 in Manitoba resulted in the swift enactment of social distancing protocols in mid-March 2020. I was conducting research with Knowledge Holders where I was exploring aspects of loss and grief unique to Indigenous peoples. In-person research interactions were halted by the university. In keeping with traditional transmission of Indigenous knowledge, I informally spoke with my network of Knowledge Holders, Indigenous colleagues and friends about the notion of communal grief and how we move forward with healing in this altered reality of the global pandemic. Adding more complexity to this situation, the COVID-19 restrictions also prevented funerals, wakes and ceremonies as gatherings of more than 10 people were prohibited. The conversations within my network brought to light Indigenous knowledges that mirror the lessons of Albert White Hat. We discussed the wide range of rituals conducted by the living that help the spirit move on with their journey and not only practice but create meaning in community by coming together to keep a fire for four days, tend to and hold a vigil with the body, make offerings, pray, and hold ceremony as well as feed the spirit and each other. In sum, the community, through these protocols and practices, share the weight of the grief, with particular focus on helping the family. People gather to support each other through the sharing of stories, offerings of healing for those suffering through prayers and practices. For those of us who are able to follow these practices, they allow individuals to feel and process grief while working to find a way to lessen the burden of others. Collectively the grief is felt and healed individually through the community prayers and support while also ensuring that the spirit is sent on its journey so it can do what it needs to do.

In my relationships with Lakota elders, I witnessed loss and grief in the reservation community. The communal grief was shared, regardless of the way a community member died: through accidents, violence or natural processes. The distances on the reservations are great – the furthest communities are nearly 100 miles apart, yet it was incredible to witness how the elders attended funerals and supported families in all communities. In modern life outside the reservation, these formal and informal practices and protocols are often not shared in non-Indigenous communities. In reflection, Indigenous people have lived on this land and fostered these practices for millennia.

Indigenous communities, even in urban areas, may not share exactly the same tightly-knit cultural connections as in reservation communities, however, in Winnipeg where the Indigenous community is large but intertwined through a web of relationships, communal grief is experienced. When the news of the three deaths were shared in the media, I thought of the Lakota elders’ response to deaths on their reservation through the practice of prayer and being present for the family. When something happens to one of us – be that a tragic death or a great success, then it is felt by many of us because of our collective orientation towards our own communities and the spiritual connection we feel across Indigenous peoples in our territories, countries and continents.

For the deaths of the three Indigenous people, especially of Eishia Hudson, many Indigenous folks in Manitoba discussed the communal loss and grief for one of our children taken away too early in a preventable death at the hands of the police. From my research and these conversations, the characteristics of Indigenous communal grief include: a) an emotional response shared across Indigenous peoples, b) emotional response to a loss of our relations – any part of creation, and c) the acknowledgement of the spiritual connections and relationships among Indigenous people which fosters the shared emotional response to the losses. While there is great diversity within and among Indigenous peoples, practices vary widely; one commonality is to provide support to each other and to send the spirit on its journey in a positive way. The relatives left behind do our best to honour the traditions in spite of the obstacles related to the pandemic and police violence. Yet we are left with a range of feelings related to unmet responsibilities, inadequacy, guilt and the desire to honour the lives and allow their spirits to continue its journey. These feelings of not being able properly to have end-of-life ceremonies adds another layer of disruption to the complexities of communal and individual grief.

## Reflection

In reflecting on the recent deaths of the three Indigenous people who were killed by police in Manitoba in April 2020, the context for this situation changes frequently. There are two major global phenomena occurring contemporaneously – the COVID-19 pandemic and the U.S. Black Lives Matters (BLM), anti-racism movement. The uncertainty of changes related to the ever-evolving social and economic landscape is causing a collective experience of a wide range of losses which seems to result in grief. Complicating this further, Indigenous people are navigating historical losses, contemporary losses, feeling communal grief of injustices and deaths of our relatives. As one Ojibwe Knowledge Holder shared, “Indigenous people are always grieving.” Being aware that Indigenous people are navigating through many complexities is essential, while also knowing there is strength in our communities and in the healing of communal grief.

Social work has a major role to play in addressing the causes of Indigenous communal grief. While Indigenous people are proportionately a small population, we are often overlooked even though we also face high rates of racism and oppression, including higher rates of deaths by the police. Macro-social workers are working within communities to raise awareness, advocating and organizing to change policies, seeking justice. Further work needs to focus on dismantling settler colonial structures that propagate inequality and inferiority of Indigenous people – one of the root causes of the disparities and racism in social systems. To do so, social workers can take an anticolonial stance (Hart, 2009), a social, political and cultural positioning in elevating and honouring Indigenous practices by collaborating with Indigenous peoples and centering their values, practices and needs to create systems build on localized Indigenous worldviews – much like the MSW-IK program – need to happen more often.

Social workers can also recognize the importance of grieving as a community and that these practices are not confined by western notions of death and funerals. At the same time, the COVID-19 pandemic will be a persistent condition for the foreseeable future. It is imperative that the social restrictions of the pandemic are not used a tool to oppress the cultural needs of Indigenous peoples. Social workers can use their skills to work against these forces to assist in following both the health guidelines and Indigenous cultural practices. One method for working within and across communities includes the recognition of the collective survival strategies that communities have always used to endure, heal and rebuild from the communal losses and grief (Bell et al., 2019). This may include gathering to grieve collectively while also fighting for justice on behalf of a loved one, as Eisha Hudson’s family continues to publically organize. At these gatherings, Jason Collins and Stewart Andrews are also invoked and remembered. Hudson’s family leads the community in forging paths of healing and continuing the ceremony of sending her spirit on its journey. Another strategy to support the healing for Indigenous peoples is to remember our philosophies and spiritual practices have allowed Indigenous people to endure, heal and rejuvenate as we addressed the many communal losses experienced over the last few centuries.
